# Supplementary material for: Enhanced Age-Dependent Motor Impairment in Males of Drosophila melanogaster Modeling Spinocerebellar Ataxia Type 1 Is Linked to Dysregulation of a Matrix Metalloproteinase
Source: Biology (Basel). 2024 Oct 23;13(11):854. doi: 10.3390/biology13110854 (PMC11591802; doi:10.3390/biology13110854)

Supplementary Information

Supplemental Table S1: Primer sequences of genes used for qRT-PCR in this study.

Gene	Forward primer (5'-3')	Reverse primer (5'-3')
hATXN1	GAT CGA CTC CAG CAC CGT AG	GAT GAC CAG CCC TGT CCA AA
dMMP1	CCA GTT CGG CTA TCT ACC CG	CTC GAT GGC ACT CAC CCA G
dMMP2	GAA ATC GGC TCC AAT GTG CG	GCT CCA CGT AAG ATC CGT TCT G
dTIMP	AAG CAT TTG GGT TTA TTG ACG C	GTC TGT GGG TGA GAT GGC A
dHh	CGC TGG TCC TCA AGC AGA C	GAT CAC ACC CTC CAG AGG TC
dbnl	AAT GTC GCC CGC TGA CAA TA	TTG CTG ATG GGC GTG TTA CT
dSMN	CTC CGC TAT TTG GGC TAT GAG	GGA GTC TTT GAA TGA CTA CCA GC
per	GGC ACA GAG CTT TCC GAT TC	CTC CCA GGT CTT GTC GAA CT
tim	ATG GAC TGG TTA CTA GCA ACT CC	GGT CCT CAT AGG TGA GCT TGT
Clk	TCC TCA GCA ATG ACG AAT ACA C	AGG TAA TAC TTT CCG AGG CGT A
cyc	TCC GAT TAC CGG CCT AGC TT	ACG AAC AGG AAT CCC TCC GA
rp49	CAG TCG GAT CGA TAT GCT AAG GTG T	TAA CCG ATG TTG GGC ATC AGA TAC T

Supplemental Table S2: Data representing the percentage of rhythmic flies (based on number of rhythmic individuals out of total number of flies tested for each genotype and age), the period of rhythm (in constant darkness – DD) and the number of individuals of a genotype tested. Flies of each age (young or old) from each genotype were tested separately. Period of rhythm is represented as mean (hours) ± SD.

Age	Genotype	% Rhythmic	Period of rhythm in DD (Hrs)	Sample size (*n*)
**Young ~ 5 days**	***elav-GAL4/+***	100	23.6 ± 0.2	48
	UAS-ATX1.82Q/+	98	23.4 ± 0.1	42
	elav>ATX1.82Q	67	24.2 ± 0.3	64
**Old ~ 30 days**	***elav-GAL4/+***	57	24.2 ± 0.2	32
	UAS-ATX1.82Q/+	55	24.3 ± 0.6	38
	elav>ATX1.82Q	23	24.5 ± 0.4	42

Supplemental Video S1: Negative Geotaxis (RING) assay of 30 day old Control flies (elav-GAL4/+ and UAS-ATX1.82Q/+).

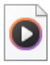

30 Day old Control flies.mp4

Supplemental Video S2: Negative Geotaxis (RING) assay of 30 day old SCA flies (elav>ATX1.82Q).

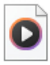

30 Day old SCA1 flies.mp4

Supplemental Figure S1: Sample full blot of dMMP1 in control (C): elav-GAL4/+ and UAS-ATX1.82Q/+ and SCA1 (elav>ATX1.82Q) flies

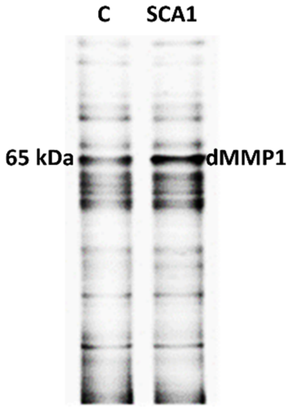

Supplemental Figure S2: Relative mRNA expression of *hATX-1* (encoding for hATX1.82Q) was significantly elevated in 5 day and 30 day old SCA1 flies (elav>ATX1.82Q) compared to 5 day and 30 day old control flies (elav-GAL4/+ and UAS-ATX1.82Q/+) which did not show expression of *hATX-1* (only symbols visible with no bar). Relative expression in 30 day old SCA1 flies was thus calculated by setting the expression levels in 5 day SCA1 flies at 1.An age-associated increase in *hATX-1* expression was recorded in SCA1 flies. Data represent mean ± SD (n=6). Bars with different superscripts are significantly different at $p<0.05$. Data were subject to one-way ANOVA with Bonferroni's post-hoc test.

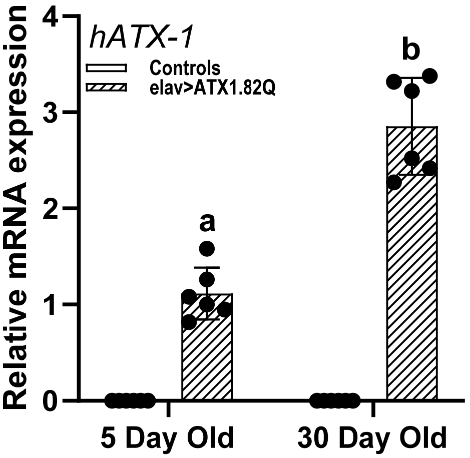

Supplemental Figure S3: Daily mRNA profiles of *Period* (*per*), *Timeless* (*tim*), *Clock* (*Clk*) and *Cycle* (*cyc*) in heads of 30 day old control male flies (elav-GAL4/+ and UAS-ATX1.82Q/+) and SCA1 male flies (elav>ATX1.82Q). ZT represents Zeitgeber time, where ZT 0 is lights on (9 am) and ZT 12 is lights off (9 pm). Expression was normalized to trough (ZT4) values set at 1 for *per*, *tim* and *cyc* and ZT 16 for *Clk* for each genotype. White and black horizontal bars mark periods of light and dark, respectively. Each data point represents mean ± SEM for three independent RNA samples. Statistical significance between differences in expression levels was tested using unpaired t-test and is denoted by ***p<0.001 and **p<0.01.

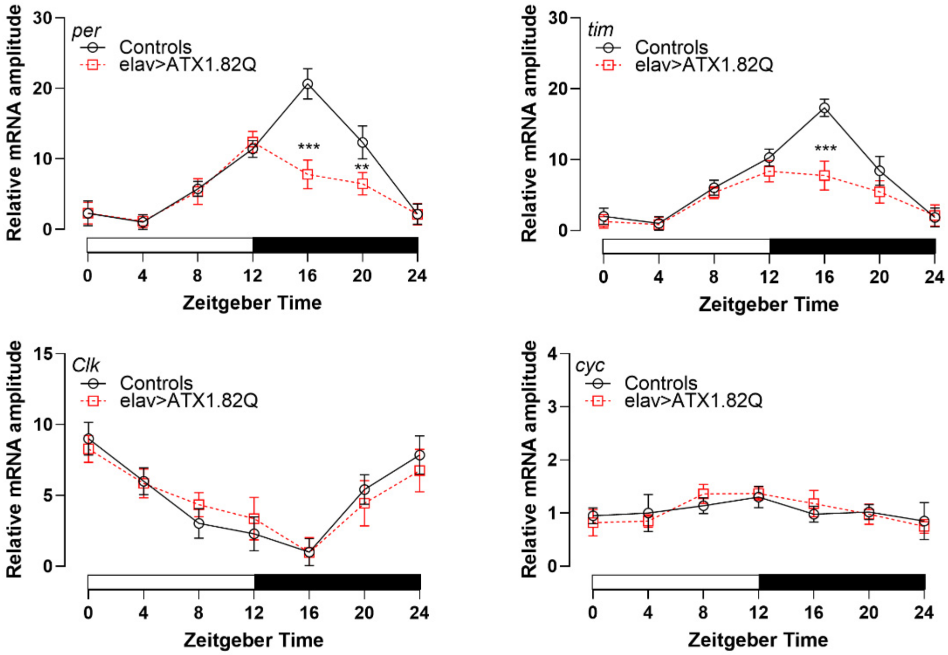

Supplemental Figure S4: Relative mRNA expression of *dbsk* (encoding for c-Jun Kinase) was significantly elevated in 5 day and 30 day old SCA1 flies (elav>ATX1.82Q) compared to 5 day and 30 day old control flies (elav-GAL4/+ and UAS-ATX1.82Q/+). Also, an age-associated increase in *dbsk* expression was recorded in both genotypes. Data represent mean ± SD (*n*=6). Bars with different superscripts are significantly different at $p<0.05$. Data were subject to one-way ANOVA with Bonferroni's post-hoc test.

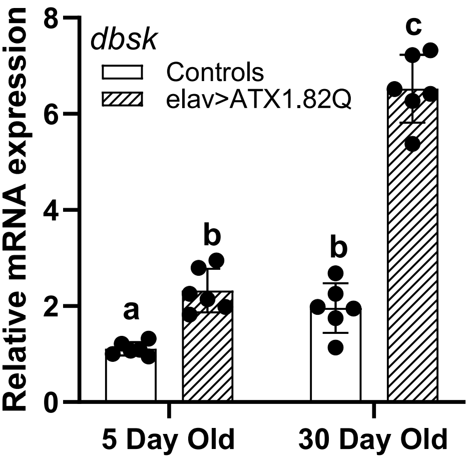

Supplement: Supplementary file 1 [file biology-13-00854-s001.zip › biology-3270517-supplementary.pdf]
